# How I do it: microvascular decompression for classical trigeminal neuralgia by arterial compression

**DOI:** 10.1007/s00701-026-06957-6

**Published:** 2026-06-27

**Authors:** M. Sindou, C. Son, A. Brinzeu

**Affiliations:** 1 University of Lyon, Lyon, France; 2 Timisoara Neuroscience Research Centre, Department of Neurosciences, University of Medicine and Pharmacy “Victor Babes” Timisoara, Timișoara, Romania; 3 Centre d’Etude et de Traitment de la Douleur, ELSAN, Clinique Bretéché, Nantes, France

**Keywords:** Classical trigeminal neuralgia, Microvascular decompression, Cerebellopontine angle arteries, Neurovascular conflict, Vascular compression syndrome, Cranial nerve disorders

## Abstract

Microvascular decompression has been shown in multiple long-term series to provide durable pain freedom in 75–95% of patients with classical trigeminal neuralgia, defined as neuralgia related to an identified neurovascular conflict, frequently arterial. Surgical success relies on an effective decompression, achieved by displacing the offending arteries while avoiding neo-compression(s) by implanted material. The procedure is performed through a keyhole retrosigmoid (retromastoid) craniectomy, using an infratentorial, supracerebellar corridor to the trigeminal root that minimizes traction on the facial–vestibulocochlear complex. In this video, we demonstrate the step-by-step technique as applied in patients in whom high-resolution MRI demonstrated the conflict preoperatively.

**Supplementary Information:**

The online version contains supplementary material available at 10.1007/s00701-026-06957-6.

## Introduction

Trigeminal neuralgia (TN) is one of the most common craniofacial pain syndromes and comprises several forms according to underlying aetiology [5, 10]. Classical TN is defined as neuralgia related to a demonstrable neurovascular conflict affecting the trigeminal root, often of arterial origin [3, 5]. Accordingly, microvascular decompression (MVD) has become the preferred surgical option in medically refractory classical TN, with the outcome of multiple large series beying a key element to support its role as a function-preserving, anatomically conservative procedure [1, 6, 8].

In properly selected patients, long-term outcomes show pain freedom in 75–95%, particularly when imaging demonstrates a high-grade conflict with clear displacement or indentation of the root [7, 8]. Surgical success depends on (i) rigorous preoperative imaging–based selection, (ii) a well-defined approach providing safe access to the trigeminal complex and minimizing risk, and (iii) complete decompression of the entire root, from the pons to the entry into Meckel’s cave (porus trigeminus), achieved preferentially by mobilizing and transposing the offending vessel and without neo-compressions by the prosthetic material [4, 9].

Alternative treatments, including percutaneous ablative procedures and stereotactic radiosurgery, are reserved for idiopathic and selected secondary forms of TN, or for patients unfit for open surgery [2, 3, 10]. The step-by-step technique demonstrated here for arterial compression by either the Superior Cerebellar Artery (SCA) or the Antero-Inferior Cerebellar Artery (AICA) corresponds to the method used in the senior author’s outcome studies [8, 9].

## Relevant surgical anatomy

Anatomically sensory trigeminal fibers course from the ganglion to the trigeminal root entry zone (TREZ) at the level of the pons. From the surgeons’ point of view in the cerebello-pontine-angle the root emerges anterolaterally and courses medial-to-lateral to Meckel’s cave, beneath the tentorium and above VII–VIII, supporting an infratentorial–supracerebellar (“tentorial-axis”) corridor for TREZ-to-porus inspection for minimal VII–VIII manipulation.

**Fig. 1:**
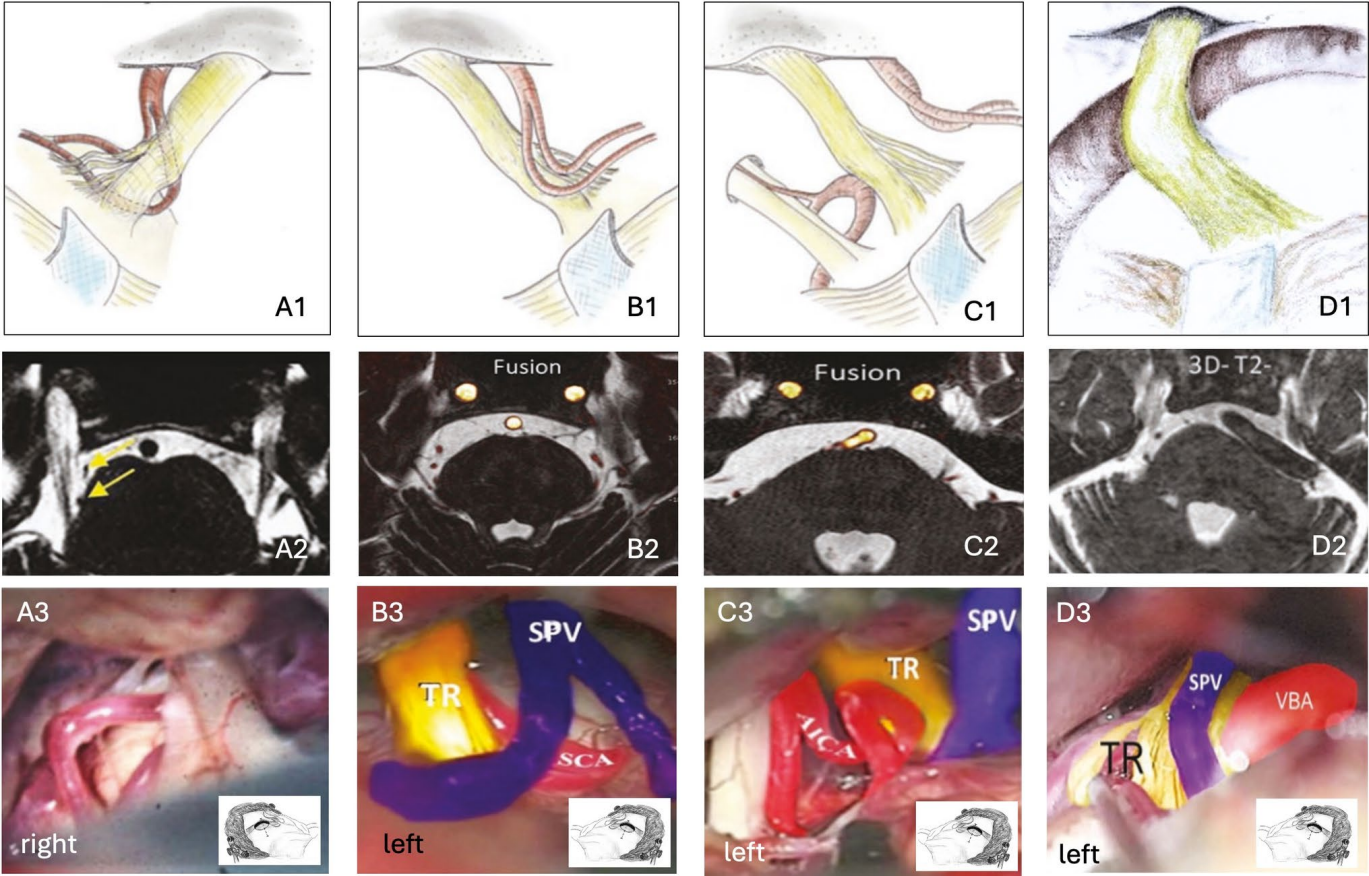
Main neurovascular conflicts patterns from arterial origin: schematic–MRI–intraoperative correlation. Panels are arranged as four vertical series (**A**–**D**), each illustrated by: (1) a schematic drawing of the conflict, (2) the corresponding high-resolution T2 MRI, and (3) the matching intraoperative view. Conflicts are graded for severity from 1 to 3 with Grade I representing simple contact, Grade II representing displacement and Grade III representing indentation or grooving of the nerve. A1-A3 (superomedial SCA, Grade II conflict right-sided TN): The SCA and its two branches impact the trigeminal root on its superomedial aspect, a frequent configuration that typically defines the initial operative corridor beneath the tentorium. B1-B3 (superolateral SCA, Grade III conflict, left-sided TN): Compression by SCA branches on the superolateral surface of the root. Note the superior petrosal vein (SPV), respected, in the operative view. C1-C3 (inferior AICA, Grade III conflict, left-sided TN): An AICA loop contacts the root from below, accounting for most inferior conflicts. This configuration is particularly important to recognize pre- and intra-operatively because the approach vector differs from SCA conflicts, and arterial mobility may be limited by perforators. Note SPV in the operative view. D1-D3 (VBA conflict, severe “mass-effect” pattern): A megadolichoectatic VBA produces marked compression and stretching of the trigeminal root from below; the SPV is seen running parallel to the root. Noteworthy in this case, because pure decompression was not considered possible, the decision was taken to cut the sensory rootlets corresponding to the trigger-zone, according to the somatotopic distribution within the root

Neurovascular conflict may occur at the TREZ (50%), mid-cisternal (40%) or porus (10%). Offenders are mainly arterial (SCA 88%, AICA 25%, dolichoectatic vertebrobasilar artery 5%); multiple conflicts (one-third) make total > 100%. Compression is usually superior (89.3%: superomedial 61.7%, superolateral 27.6) vs inferior (10.7%), guiding inspection and mobilization (Fig. 1).

Veins—chiefly the superior petrosal system—which cross the corridor, may hide the conflict. A superior petrosal trunk drains to the superior petrosal sinus, receiving mesencephalic, cerebellar and lateral pontine tributaries those should be preserved when feasible because sacrifice carries neurological risk.

## Description of the technique

### Preoperative planning

We “rehearse” MVD at the MRI console by localizing the neurovascular conflict, identifying the offender, predicting the approach vector, and judging whether durable separation by transposition is feasible.

Imaging protocol: (1) high-resolution 3D T2 (CISS/FIESTA/T2-DRIVE) tracks CN V from TREZ to porus trigeminus and grades compression [3, 5, 10]; (2) 3D TOF-MRA defines arterial loops or dolichoectasia; (3) contrast 3D T1 may detect (possible) venous conflicts, maps the superior petrosal venous complex draining to the superior petrosal sinus, anticipating venous constraints and bleeding risk. Fusion, 3D or diffusion adjuncts may help [7].

We offer MVD for classical TN with a clear MRI conflict, preferably grade 2–3; isolated grade-1 contact is weighed cautiously.

### Anesthesia

Surgery is performed under general anesthesia with endotracheal intubation, preferably using total intravenous anesthesia (propofol–remifentanil) to maintain hemodynamic stability and promote cerebellar relaxation.

### Operation room setup, installation and patient positioning

**Fig. 2:**
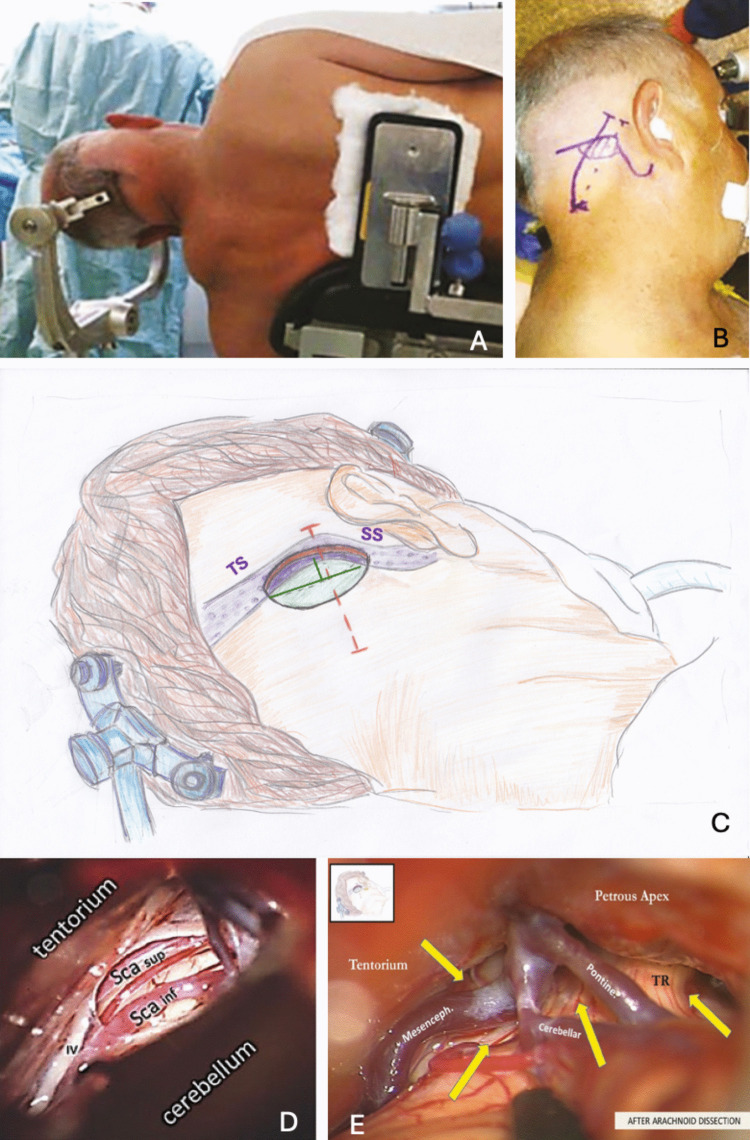
Installation and supracerebellar-infratentorial exposure of the trigeminal nerve root region. **A** Installation. The patient is positioned in lateral decubitus, the torso slightly rotated backwards so as the ipsilateral shoulder “falls” away from the operative corridor. The head is secured in a three-pin head holder (one pin above the ipsilateral eyebrow, two in occipital region with moderate elevation, slight flexion (saggital—chin kept two finger-breadths from the sternum to avoid jugular compression), and a 15° rotation (axial) toward the contralateral side; a slight contra-lateral (coronal) flexion helps prevent the shoulder from obstructing the working axis. The ipsilateral shoulder is gently taped and pulled caudally (avoiding excessive brachial plexus traction), and the head of the table is mildly elevated (10°) to facilitate venous return. The surgeon stands behind the patient; the microscope is brought over the ipsilateral shoulder and aligned toward the tentorial plane and trigeminal root region. **B** Skin landmarks. Before incision, the mastoid process (projection of the sigmoid sinus), the superior nuchal line/occipital crest (projection of the transverse sinus), and the estimated transverse–sigmoid junction are marked. The planned incision is retroauricular and oblique, 1 cm medial to the bisector of the angle of sinuses, centred to provide a “high” retrosigmoid infratentorial-supracerebellar corridor. **C** Skin incision, muscle dissection, and craniectomy. A 7-cm oblique retroauricular incision is performed, placed 1 cm medial to the bisector of the angle formed by the superior nuchal line and the posterior margin of the mastoid process. The subcutaneous tissues and muscles are divided with cautery; the occipital artery, if encountered, is ligated and divided, and the posterior mastoid region is cleared. The mastoid emissary vein—when exposed—should be obturated (gelfoam then wax). A tailored keyhole retromastoid retrosigmoid craniectomy is then fashioned: a burr hole is placed just posterior to the base of the mastoid process and just below the nuchal line, and enlarged with rongeurs to expose (1) the transverse sinus superiorly and (2) the posterior border of the sigmoid sinus laterally; the opening is typically elliptic (2 cm wide × 1.5 cm high) with the long axis parallel to the tentorium, adapted to individual anatomy. If mastoid air cells are opened (frequent), they are sealed, preferably with fat (impacted in cells). **D** Dural opening and entry into the supracerebellar infratentorial corridor. The dura is opened in a “inverted T” shape to create two small flaps: a superior flap reflected upward onto the transverse sinus and a lateral flap reflected onto the sigmoid sinus, providing a working window immediately under the tentorium. After cerebrospinal fluid release to obtain cerebellar relaxation, a self-retaining retractor with a thin, malleable blade is placed gently on the superior surface of the cerebellum to develop a lateral infratentorial–supracerebellar trajectory. The microscope is oriented with its visual axis parallel to the superior petrosal sinus. Under high magnification, the prepontine – cerebellomesencephalic arachnoid is opened sharply, parallel to the tentorial incisura and approximately 2 mm inferior to the trochlear nerve (CN IV). This opening exposes the superior cerebellar artery in the peripeduncular region while maintaining a thin veil of arachnoid posterior to CN IV to reduce the risk of injury (often by with the aspiration canula). **E** Arachnoid dissection, venous management, and exposure of the trigeminal root nerve. Through the infratentorial–supracerebellar route, the superior petrosal venous system—classically located posterior to the trigeminal root—is progressively freed from surrounding arachnoid, taking care to preserve the main superior petrosal trunk and its principal tributaries (mesencephalic, cerebellar, and pontine affluent). Meticulous arachnoid dissection around these venous structures allows downward relaxation of the cerebellum without tearing the venous trunk. Only limited arachnoid opening is performed toward the facial/cochleo-vestibular nerves. The retractor tip is then adjusted to open the cerebellomesencephalic and cerebellopontine fissures, creating successive microsurgical corridors (“working triangles”). This stepwise arachnoid work permits full inspection of the trigeminal root—from its brainstem entry zone to the porus trigeminus of the Meckel’s cave. At the completion of this exposure, the trigeminal root and its surrounding venous structures are fully visualized and ready for assessment of neurovascular relationships

We perform the procedure in the lateral decubitus position **(**Fig. 2A and B**)**

### Exposure

Incision, soft-tissue dissection, retromastoid keyhole retrosigmoid craniectomy and dural opening are detailed in Fig. 2C. After dural opening, an infratentorial–supracerebellar corridor is followed to the trigeminal root using a trajectory following the superior cerebellar surface (i.e. no lateral to medial retraction) (Fig. 2D). Arachnoid is widely opened to explore the root from brain stem to porus trigeminus.

On access to the root, the superior petrosal venous system (SPVC) may constitute obstacle; preservation is preferable due to significant risk of venous infarction (Fig. 2E).

### Decompression

Decompression aims at durable separation without introducing new compression [9].

**Fig. 3:**
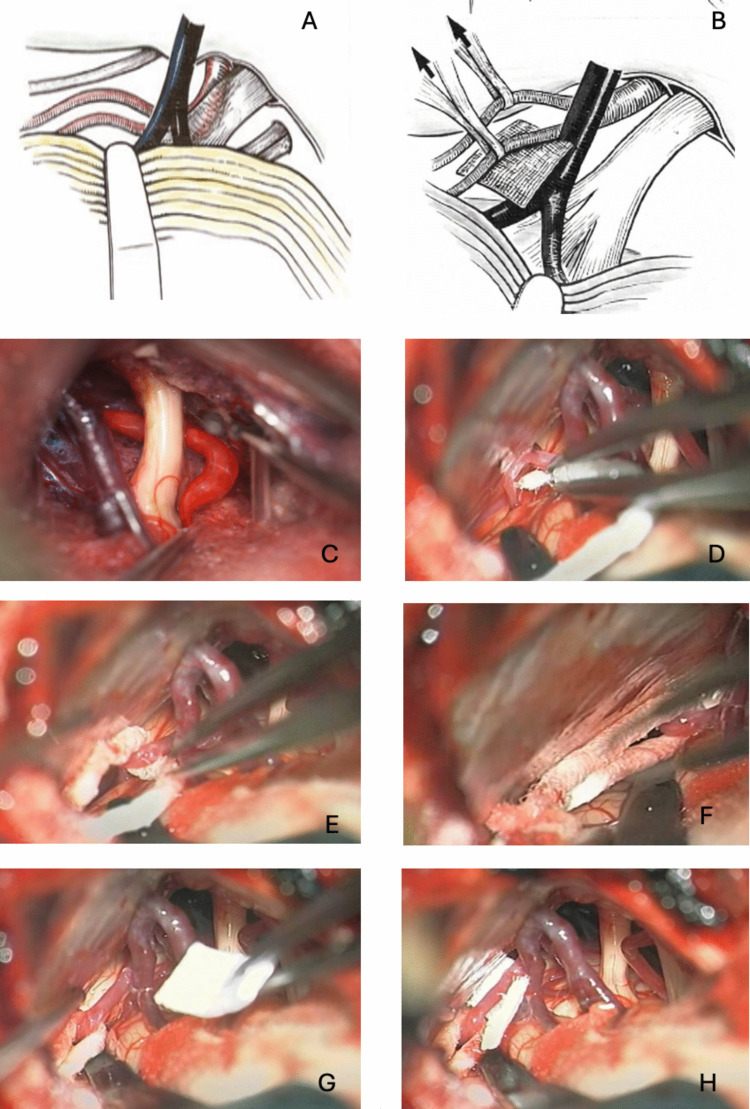
Stepwise arterial transposition (“sling” technique) for superior cerebellar artery (SCA) conflict through the infratentorial–supracerebellar corridor. After an inverted-T dural opening, the main dural flap is reflected cranially along the inferior border of the transverse sinus to expose the tentorium, and a smaller triangular flap is suspended laterally along the sigmoid sinus. The working corridor is developed by following the superior cerebellar surface (along the axis of the superior petrosal sinus) beneath the tentorium toward its free edge; cerebellar descent is directed downward (not lateral-to-medial) to avoid stretching the cochleo-vestibular nerves. **A** Intradural development of the supracerebellar infratentorial corridor (step-by-step). After the dural opening, downward cerebellar retraction is applied using a self-retaining system—preferably with narrow, malleable Sugita type blades—to reach the free edge of the tentorium. At this point, an arachnoid veil typically covers the SCA and the trochlear nerve (CN IV). The arachnoid is sharply incised and the opening is carried laterally toward the superior petrosal venous complex (SPVC) and the trigeminal root (CN V). Dissection is then performed around CN V and around the SPVC to allow inspection of the trigeminal root along its entire cisternal length (from the root entry zone to the porus trigeminus). **B** Schematic of arterial transposition (“sling”) and anti-neo-compression buttress. The SCA is transposed superiorly using a PTFE (Teflon®) sling; the sling is then wedged in the subtentorial space between the superior cerebellar surface and the tentorium to secure the transposed artery. Additional stabilization is provided by a semi-rigid knitted PTFE plate (e.g., Edwards Outflow Tract knitted PTFE) interposed between the transposed SCA and the SVC, keeping prosthetic material away from CN V and thereby avoiding neo-compression. Shredded PTFE Teflon® felt is prepared by teasing its fibers to create ribbons/balls (used here rather than the sling); polyester (Dacron) is used only rarely. **C** Intraoperative view after arachnoid opening showing a superomedial SCA–CN V conflict prior to mobilization. **D–F** Progressive steps of transposition: sharp circumferential subarachnoid dissection releases arachnoid “tethers” around the artery and root; the SCA is mobilized (superiorly); the PTFE sling is positioned to pull the artery upward and wedged beneath the tentorium, maintaining this vector while preserving the SPVC. **G–H** Final configuration: the SCA is stabilized in the subtentorial space; a knitted PTFE plate (≈5 × 5 mm) is placed between the transposed SCA and the SPVC to maintain the new arterial position while preventing neo-compression. SCA spasm, if happens, may be managed with warm irrigation and, if needed, topical droplets of papaverine. Throughout, standard CPA microsurgical instruments are used (fine bipolar forceps, microdissectors, microscissors, and suction). Exposure is maintained with minimal cerebellar retraction using a self-retaining system; we favor narrow, malleable Sugita blades to keep stable visualization without obstructing the working corridor

Anatomy permiting, arterial transposition is preferred. Usually the superior cerebellar artery (SCA) can be dissected and mobilized superiorly using the *sling* technique (Fig. 3). This is then stabilized in the subtentorial space; so without creating neo-compression on the trigeminal root.

**Fig. 4:**
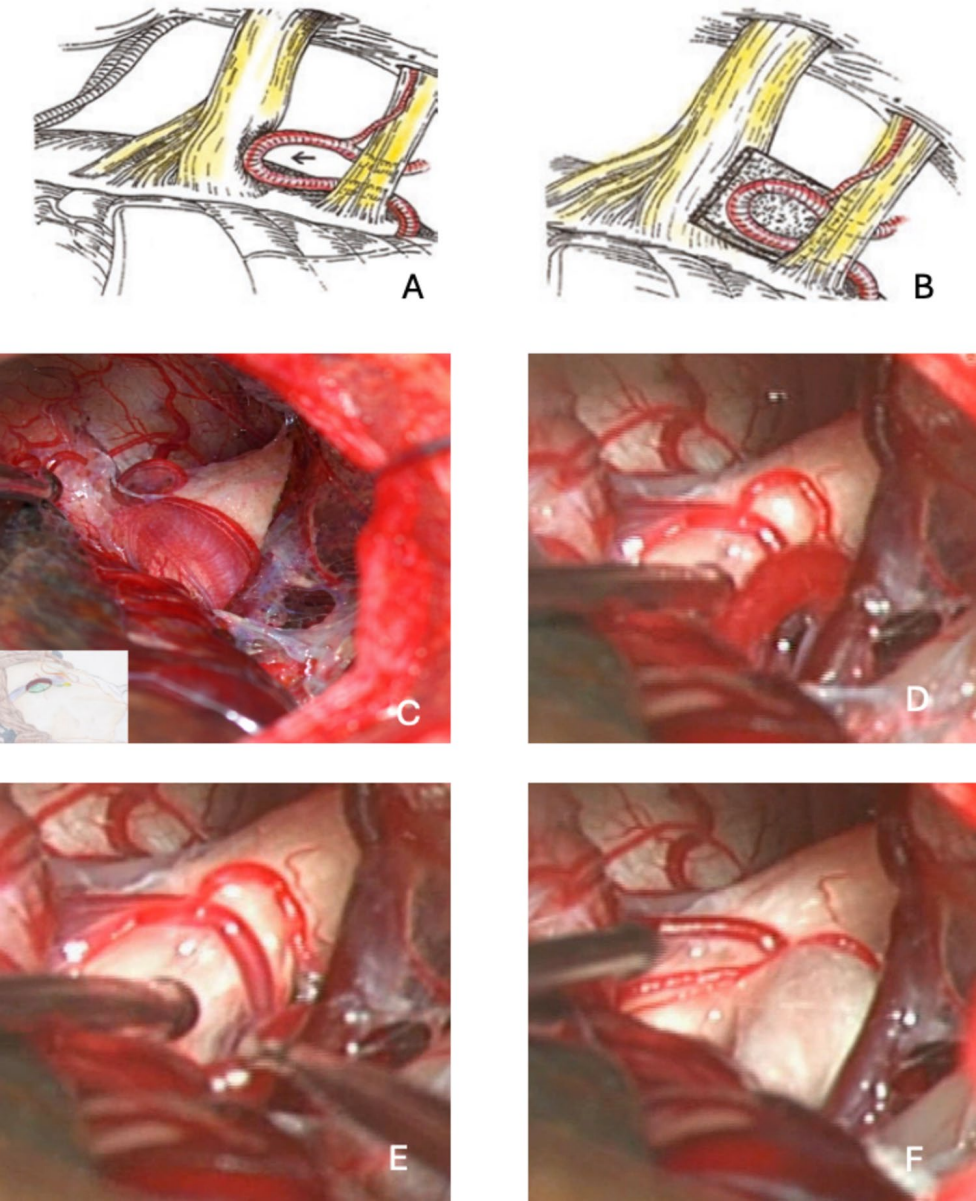
Inferior AICA conflict at the trigeminal root entry zone treated by interposition (right-sided TN). After dural opening, the AICA (anterior inferior cerebellar artery) is identified contacting the trigeminal root from below, at the TREZ. In this configuration, if arterial transposition is limited by short mobility and perforating branches—decompression is achieved by limited PTFE (Teflon) interposition using the “bridging technique”. **A** Schematic of an inferior AICA conflict on the right trigeminal root at the TREZ. **B** Schematic of decompression by Teflon interposition. **C** Intraoperative view: AICA loop compressing CN V from the inferior aspect at the TREZ. **D** Mobilization attempt demonstrating restricted transposition due to perforators/limited slack. **E** Sharp arachnoid dissection releasing adhesions around CN V and the arterial loop to define the true plane of conflict. (F) Final step: small PTFE (Teflon) pledget interposed between AICA and the nerve (without overpacking) to abolish direct pulsatile contact

AICA conflicts are less amenable to transposition because of frequent perforator constraints (Fig. 4).

When transposition is not feasible, decompression is achieved by interposing small PTFE (Teflon) pledgets. Overpacking should be avoided. No ablative procedure on the trigeminal nerve is performed, as relief of vascular compression alone is sufficient.

After decompression, inspection is performed to confirm separation from all offenders and a tension-free, non-kinked course of transposed arteries. Spasm is managed with warm saline and, if needed, topical dilute papaverine. Hemostasis is verified by Valsalva manoeuvres and/or jugular compression.

### Closure

Watertight dural closure is performed, primary suturing augmented with a small autologous graft, sealed with fibrin glue. Reconstruction of the bony defect is usually unnecessary; bone dust packing is sufficient. Compressive sterile dressing is useful in preventing CSF leaks (pseudo meningocele).

### Postoperative care

- Includes neurosurgical examination, with particularity auditory and corneal evaluation (and protection)
- Pain relief is generally immediate; anticonvulsant therapy is maintained at half-dose, gradually tapered.
- Discharge is usually after 4–8 days, check for CSF leakage.

## Indication

- Classical TN with a clear (or at least likely present) NVC on high-resolution MRI.
- Failure or intolerance of adequate medical therapy.
- Secondary neuralgias are in principle excluded.
- Patient medically suitable for posterior fossa surgery

## How to avoid complications

- Maximise the reduction of venous pressure by head elevation, limiting end expiratory, pressure and excessive head rotation to prevent swelling
- Design craniectomy and dural opening to access trigeminal root via an infratentorial—supracerebellar trajectory, to avoid stretching the CN VII-VIII by lateral cerebellar retraction
- Use sharp arachnoid dissection to fully expose the trigeminal root from brainstem to porus trigeminus, not to miss multiple conflcits.
- Preserve major veins, whenever possible to avoid venous infarction.
- Decompression should avoid neo-compression
- Watertight dural closure and mastoid cell sealing to prevent surgical site complications (CSF leakage)

## Patient information

- Rationale for MVD,
- Main procedure-specific risks:
  - cerebrospinal fluid leak
  - vascular complications.
- Alternative treatments:
  - percutaneous lesioning procedure,
  - stereotactic radiosurgery,

## Conclusion

By directly eliminating the underlying neurovascular conflict and avoiding neural injury, MVD is the definitive surgical treatment for medically refractory classical TN, offering durable pain relief with preservation of neurological function.

## 10 Key points summary

1. Classical TN is clinically diagnosed and MRI confirmed when NVC is identified
2. The treament goal for classical TN is durable vessel–nerve separation without neo-compression.
3. Expose CN V fully: follow root from TREZ to porus trigeminus and circumferentially; consider possible multiple conflicts
4. Work within the SPVC corridors: identify tributaries early; preserve when feasible.
5. Preferred strategy: arterial transposition whenever anatomy permits.
6. SCA most often: free arachnoid tethers and mobilize superiorly
7. Stabilize transposition: buttress with a Teflon plate, not against CN V.
8. AICA conflicts: mobilize, when possible, transpose inferiorly (limited by perforators).
9. If no transposition: small PTFE pledgets only; never overpack;
10. Final checks: confirm all offenders cleared, arteries tension-free/no kinking; treat spasm (warm saline and/or droplets of papaverine) and secure hemostasis; no trigeminal lesioning.

## Supplementary Information

Below is the link to the electronic supplementary material.

ESM 1 Supplementary Material 1 (MP4 429 MB)

## Data Availability

No datasets were generated or analysed during the current study.
